# Remote control of wildtype zebrafish optomotor response with a photoswitchable drug

**DOI:** 10.3389/fnbeh.2026.1916443

**Published:** 2026-10-01

**Authors:** Luisa Camerin, Joaquín Martínez-Tambella, Gregor Schuhknecht, Vickie M. Wang, Kumaresh Krishnan, Paula Pflitsch, Florian Engert, Pau Gorostiza

**Affiliations:** 1 Institute for Bioengineering of Catalonia, Nanoprobes and Nanoswitches, Barcelona, Spain; 2 Network Biomedical Research Institute in Bioengineering, Biomaterials, and Nanomedicine (CIBER-bbn), Madrid, Spain; 3 University of Barcelona, Barcelona, Spain; 4 Faculty of Arts and Sciences, Harvard University, Department of Molecular and Cellular Biology, Cambridge, MA, United States; 5 Department of Neurobiology, Harvard Medical School, Boston, MA, United States; 6 Catalan Institution of Research and Advanced Studies, Barcelona, Spain

**Keywords:** circuit perturbation, NaV voltage-gated sodium channels, neuromodulation, optogenetics, optomotor response, photopharmacology, photostimulation, sensorimotor behavior

## Abstract

**Introduction:**

For animals to interact effectively with their environment, the brain must integrate sensory information and generate appropriate motor responses. Multiple neuronal circuits contribute to this process, and identifying their roles remains a central focus in neuroscience. In this context, photopharmacology provides light-controllable compounds that enable remote and spatially and temporally precise modulation of neuronal activity without requiring genetic modification. This approach minimizes off-target activation of unwanted brain regions while allowing reversible control of neural circuits. The recently developed photoswitchable compound Carbadiazocine has been shown to reversibly control neuronal firing across multiple species in a light-dependent manner. It modulates larval zebrafish locomotion and alleviates neuropathic pain in rodents in a reversible, light-induced manner. Given the established *in vivo* effects of this compound, we further investigated its impact on sensorimotor behaviors.

**Methods:**

We focused on the optomotor response in zebrafish larvae and assessed its potential as a tool for circuit perturbation and behavioral analysis, for the first time combined with photopharmacology. We performed experiments in head-fixed and free-swimming larvae to assess their capacity to detect and follow optic flow, and to characterize swimming speed patterns and tail bout properties following administration of the two Carbadiazocine photoisomers.

**Results:**

In both paradigms, treatment with the pre-illuminated compound decreased accuracy in responding to optic flow (correct turning percentage dropping from ~95% to ~80% in head-fixed experiments and from ~65% to ~20% in free-swimming experiments). Speed analysis revealed more frequent and prolonged fast movements, with fewer slow movements, even without visual stimulation. Tail bout analysis showed increased 15–30 Hz bout frequencies, corresponding to irregular tail movements. These effects were absent with the dark-relaxed compound.

**Discussion:**

Together, these findings provide a foundation to probe neuronal circuits underlying behavior in diverse animal species using a dynamic range of photostimulation patterns.

## Introduction

Photopharmacology is an expanding field that develops spatiotemporally-controlled pharmacological compounds. Small-molecule drugs can be designed to undergo conformational changes upon light irradiation with specific wavelengths, which alter their biological effects (Velema et al., 2014; Hull et al., 2018; Broichhagen et al., 2015; Kobauri et al., 2023). This isomerization process is reversible with time by relaxation back to its thermally stable state or upon irradiation with a second wavelength. The possibility of turning on and off the biological activity of chemical compounds with specific wavelengths allows to constrain their activity within tissue using illumination patterns. Preventing unwanted activity outside the region of interest thus reduces potential adverse effects (Frank et al., 2020; Gutzeit et al., 2021; Hull et al., 2018; Gomila and Gorostiza, 2022). Such drug-based control of cellular activity with light can be exerted on all wildtype organisms expressing the target protein and does not require genetic manipulation. Thus, it is generally advantageous over optogenetic methods like channelrhodopsin overexpression.

The choice of suitable animal models is essential to study photoactivation inside an organism. Zebrafish (*Danio rerio*) has proven valuable to test and screen photoswitchable drugs (Gomila and Gorostiza, 2022; Silva et al., 2025; MacRae and Peterson, 2015; Matera et al., 2018; Prischich et al., 2021; Matera et al., 2022; Sortino et al., 2023; Camerin et al., 2024; Prischich et al., 2024). Zebrafish are freshwater tropical fish in the Cyprinidae family. They share approximately 70% of their genome with humans and 84% of human disease-associated genes have zebrafish homologs (MacRae and Peterson, 2015; Barbazuk et al., 2000; Howe et al., 2013). Its small size and rapid development make it a promising model for studying human disease, neuronal functions, and development (Kimmel et al., 1995; Kimmel et al., 1982; Ober et al., 2003; Field et al., 2003; Robertson et al., 2007; Stewart et al., 2014). The fact that larvae are naturally translucent for the first few weeks of life facilitates observation of organs and internal structures under the microscope in living organisms and in real time, as well as the capability for modulating the activity of photoswitchable drugs. This makes zebrafish larvae a versatile model for a wide variety of photopharmacological studies. Larvae have been used for toxicity and efficacy studies with several readouts, including survival rate, developmental abnormalities, heart rate modulation, tumor growth control, and visual acuity (MacRae and Peterson, 2015; Stewart et al., 2014; Feitsma and Cuppen, 2008; Huang, 2008; Sortino et al., 2026). Swimming activity is generally used to evaluate not only the safety profile of drug candidates, but also to assess their effects on targets such as dopaminergic or glycinergic neuronal circuits that regulate locomotion (Matera et al., 2022; Camerin et al., 2024; Burton et al., 2021; Gómez-Santacana et al., 2017; Prischich et al., 2021). However, these assays are often limited to quantifying whether a drug increases or decreases locomotion, and complementary experiments are required to elucidate the drug action site(s) in the neural pathway and whether other neural processes are being modulated. Among the wide repertoire of behavioral tests that can be implemented on zebrafish larvae, those combining complex sensory stimuli with specific locomotor readouts offer an added value to the standard swim distance quantification as they allow us to probe the whole sensorimotor process underlying the observed behavioral response (Stewart et al., 2014; Kalueff et al., 2014; Guggiana-Nilo and Engert, 2016). Moreover, the development of sophisticated tracking software for larval zebrafish has permitted the study of locomotor output with enough granularity to classify individual swim episodes according to their speed and duration and to study motor responses at single bout resolution (Bahl and Engert, 2020; Portugues and Engert, 2009).

For these reasons, the zebrafish optomotor response (OMR) behavioral assay has been an instrumental tool to study complex sensorimotor processing and its underlying circuits (Naumann et al., 2016; Tanaka and Portugues, 2025; Fouke et al., 2025; Simões et al., 2025; Bonnen, 2023). Fish have an innate ability to detect visual motion and follow the direction of the perceived movement, which is a critical survival mechanism that is thought to help them avoid involuntary displacement in water currents and to evade predators (Budick and O’Malley, 2000; Zalocusky et al., 2013). The OMR in zebrafish is characterized by discrete swim bouts, each comprising a few tail oscillations, which can be analyzed as individual decisions resulting from sensory inputs. These bouts are interspersed with quiescent periods, known as interbouts, during which sensory information may be accumulated (Bahl and Engert, 2020; Orger et al., 2000; Neuhauss et al., 1999; Burgess and Granato, 2007; Johnson et al., 2020; Dragomir et al., 2020).

The OMR in zebrafish larvae has been widely reproduced with a stimulus pattern made of projected high-contrast parallel stripes that move perpendicular to the stripe direction in the fish arena (Maaswinkel and Li, 2003). In addition, the random dot kinematogram (RDK), a visual stimulus originally developed for primate studies, has been successfully adapted for zebrafish larvae (Bahl and Engert, 2020; Dragomir et al., 2020). It consists of projected dots moving perpendicular to the fish body axis. When exposed to whole-field motion drift from the dots, zebrafish exhibit the OMR and move in the same direction as the dots. This behavior requires no training and is present in larvae as early as 5 days post-fertilization (dpf), making it an ideal system for exploring the neural basis of sensorimotor integration and decision-making.

OMR experiments have been combined with optogenetic control of genetically modified neurons in zebrafish, allowing to map certain circuits and to investigate sensorimotor transformations (Naumann et al., 2016). However, despite the unique ability of photoswitchable drugs to control intact neurons in wildtype fish with pharmacological and spatiotemporal selectivity, OMR assays have never been performed under photopharmacological control.

Carbadiazocine is a photoswitchable analog of carbamazepine, a voltage-gated sodium channel blocker that controls neuronal firing. Addition of dark-relaxed Carbadiazocine (100% cis) to water has no effect on wildtype zebrafish larvae locomotion, while photoisomerization via irradiation with 405 nm light (50% trans) increases the distance swum (Camerin et al., 2024). Here, we have combined OMR assays in freely moving and head-fixed wildtype zebrafish larvae with the use of Carbadiazocine in its two forms. We observe that the illuminated compound decreases the accuracy of visual stimuli responses, enhances fast swimming movements over slow ones, and causes irregular tail movements. These findings lay the foundations to probe intact neuronal circuits underlying sensorimotor transformations in diverse animal species using patterns of photostimulation at wide spatiotemporal scales.

## Results

To evaluate the effect of Carbadiazocine on zebrafish behavior involving complex sensorimotor integration, we first performed OMR tests with free-swimming larvae. The experiment was designed in two consecutive parts (see Figure 1A, details in Methods). In the first part, the baseline swimming response to the OMR stimuli was assessed. In the second phase, larvae were treated with either the inactive or the active isomer of the photoswitchable compound, and their swimming responses to the OMR stimulus were re-evaluated. The results were analyzed by comparing the larvae initial and final positions within each well during the presence of the stimuli. A response was considered correct if the larva moved in the same direction of the stimuli (i.e., if the stripes moved upward in Figure 1A and the larva’s final position was higher than its initial position). In practice, a correctness threshold for larvae position was established as the mean Y coordinate variation of each untreated larvae in absence of stimuli (see Methods for details).

**Figure 1:**
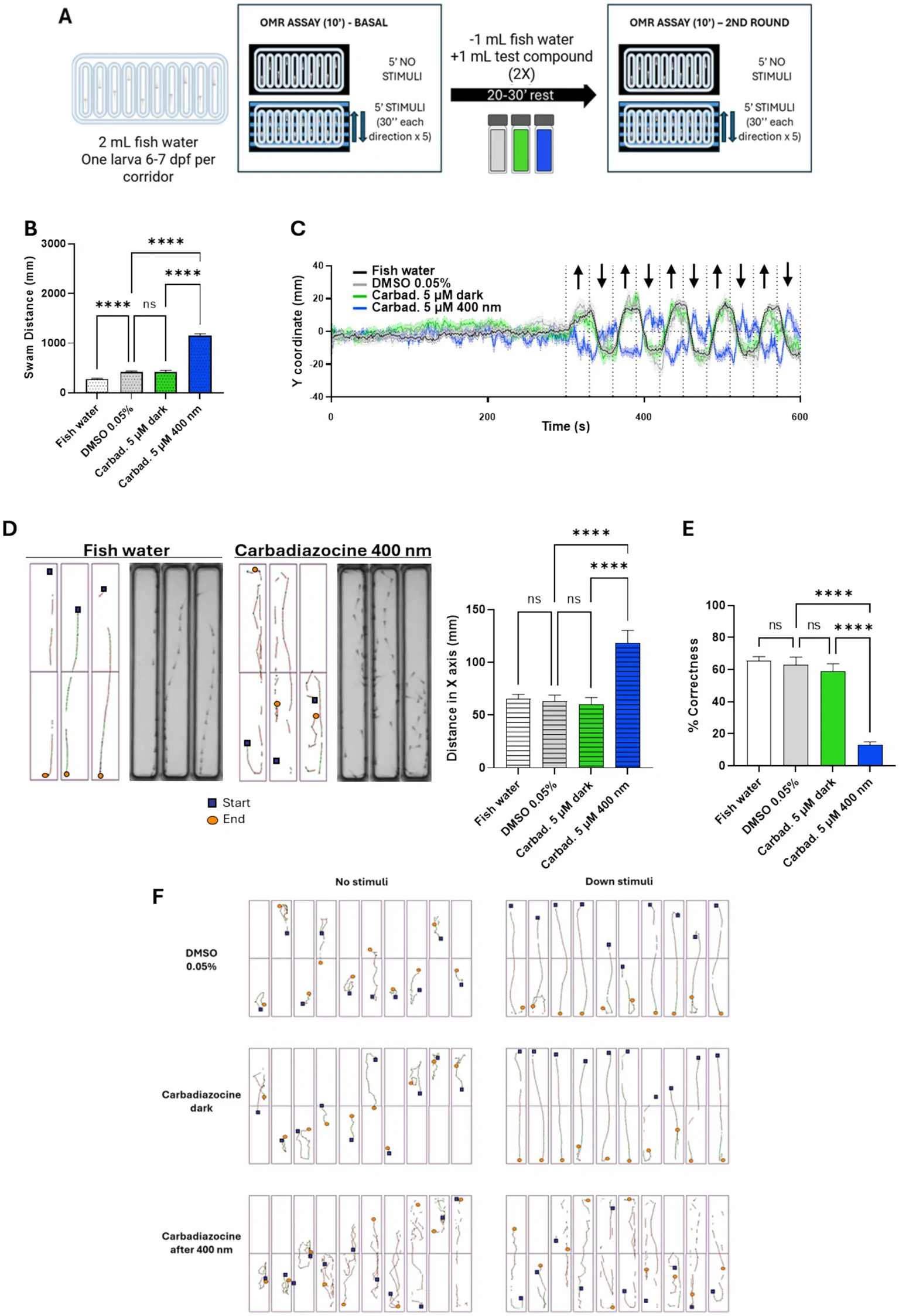
Optomotor response assay in free swimming zebrafish larvae; *n* = 40 for DMSO 0.05%, *n* = 50 for Carbadiazocine dark (100% cis) and pre-illuminated at 400 nm (50% trans) at 5 μM. **(A)** Experimental design showing 10 linear corridors for individual larvae (vertical in the figure) over a screen that projects patterns of horizontal blue and black (dark) stripes that move in the vertical direction, triggering the optomotor reflex in untreated larvae to follow them. Created in BioRender. Martínez Tambella, J. (2026) https://BioRender.com/wtmrgz9. **(B)** Total distance swam by the zebrafish larvae in the protocol period without stimuli. Carbadiazocine 5 μM preilluminated at 400 nm light significantly increased the swam distance. **** *p* < 0.0001 Mixed effect analysis with Šídák’s multiple comparisons test. **(C)** Temporal trace of the vertical (Y) position of the larvae during the experiment. The horizontal dotted line shows the central position of the plate, and vertical dotted lines indicate the change in the visual stimuli. Arrows indicate the direction of the moving stripe stimuli in each period. **(D)** Distance swam orthogonal to the stimuli (horizontal, X) and example of traces from 30-s stimuli (stripes moving down) of the same three fish before and after the application of Carbadiazocine pre-illuminated at 400 nm. Red lines correspond to fast movements (distance traveled at ≥ 6 mm·s^−1^); green lines correspond to intermediate movements (distance traveled at 2–6 mm·s^−1^); black lines correspond to slow movements (distance traveled at ≤ 2 mm·s^−1^). **** *p* < 0.0001 Mixed effect analysis with Šídák’s multiple comparisons test. **(E)** Percentage of correctness of the OMR assay. See Supplementary Figure S2 for the estimation plot. **** *p* < 0.0001 Mixed effect analysis with Šídák’s multiple comparisons test. **(F)** 30-s trajectories of zebrafish larvae swimming in the absence or presence of stripes stimuli moving downwards (left and right panels respectively) under different treatments (vehicle DMSO 0.05%, dark-relaxed Carbadiazocine and pre-illuminated Carbadiazocine). Blue squares represent larva position at the beginning of the period and yellow circles their final position.

Untreated larvae (basal) swam relatively short distances, mainly displacing along the wells in the direction of visual stimuli (Y or vertical in Figure 1C) and displayed high correctness (Figure 1E), consistent with zebrafish OMR (Fleisch and Neuhauss, 2006). Although the vehicle (0.05% DMSO) increased the swim distance in absence of stimuli, no further increase was observed with the dark-relaxed Carbadiazocine, confirming that the *cis* relaxed isoform of the drug does not alter larvae locomotion (Camerin et al., 2024; Figure 1B). In contrast, larvae treated with Carbadiazocine pre-illuminated at 400 nm showed a two-fold increase in swim distance (Figure 1D) and a three-fold decrease in correctness (Figure 1E). The effect of the active isoform of the drug was evident immediately upon administration, even before visual stimulus onset, because the larvae exhibited non-directed hyperactivity (Figure 1F). In presence of the visual stimulus, hyperactivity persisted and the larvae continued to modify their swimming direction each time the OMR stimulus changed (up or down), albeit in the opposite direction to the stimulus in the case of Carbadiazocine pre-illuminated at 400 nm (Figure 1C; Supplementary Figure S1; Supplementary Movies 1, 2). Moreover, these treated larvae presented a significant increase in the distance swam orthogonal to the stimuli (X direction, Figure 1D). The hyperactivity observed in absence of moving stripes, along with the changes in swimming direction and behavior in response to visual stimuli, could be due to the drug interfering with a motor circuit (e.g., producing an under/overshoot). Interestingly, such interference does not impair the fish’s ability to detect the presence of visual stimuli in their environment.

The observed results regarding hyperactivity are consistent with the action of Carbadiazocine on the swimming behavior of both blinded and non-blinded zebrafish in circular wells (Camerin et al., 2024). The effects of the drug isomers on the correctness of movements (time course and direction of OMR trajectories) have not been previously reported and provide new insights for circuits perturbation tools. In particular, they show that correctness can be controlled with pharmacological selectivity and offer exciting prospects of spatial and temporal selectivity by using suitable light patterns (e.g., to photoswitch Carbadiazocine in the entire central nervous system, in selected groups of neurons, in individual ones, or even in specific subcellular regions like axon hillocks or nodes of Ranvier). This convenient manipulation parameter was not previously available in advanced behavioral studies like OMR that use naturalistic stimuli to evoke innate behaviors in wildtype individuals.

To better understand the characteristics of larvae movement in the different conditions, we quantified the speed of each movement event and classified them as fast (> 6 mm·s^−1^), intermediate (2 to 6 mm·s^−1^), and slow (< 2 mm·s^−1^). During the initial no stimuli period, pre-illuminated Carbadiazocine increased the total number of movement events across all speed categories (Figure 2A). However, the duration and distance swam per movement were significantly reduced for slow movements and increased for fast movements (Figure 2B), consistent with a decreased total distance traveled at speed < 2 mm·s^−1^ and a greater distance covered with movements exceeding 6 mm·s^−1^ (Supplementary Figures S3, S4). Pre-illuminated Carbadiazocine altered the distribution of distance traveled across speed categories during the no-stimuli period, shifting larval displacement from predominantly slow exploratory movements to a swimming behavior dominated by fast bursts (Figure 2C) even in the absence of visual stimuli to guide behavior. Overall, these results suggest that Carbadiazocine may impair or prematurely interrupt slow movements —reducing each bout duration and distance— while increasing the frequency and duration of fast swims. These outcomes could result from different effects of drug isomers on the neural circuits controlling locomotor behaviors. The site(s), timing, and mechanism of action can likely be investigated using spatiotemporal patterns of drug photoswitching as discussed above.

**Figure 2:**
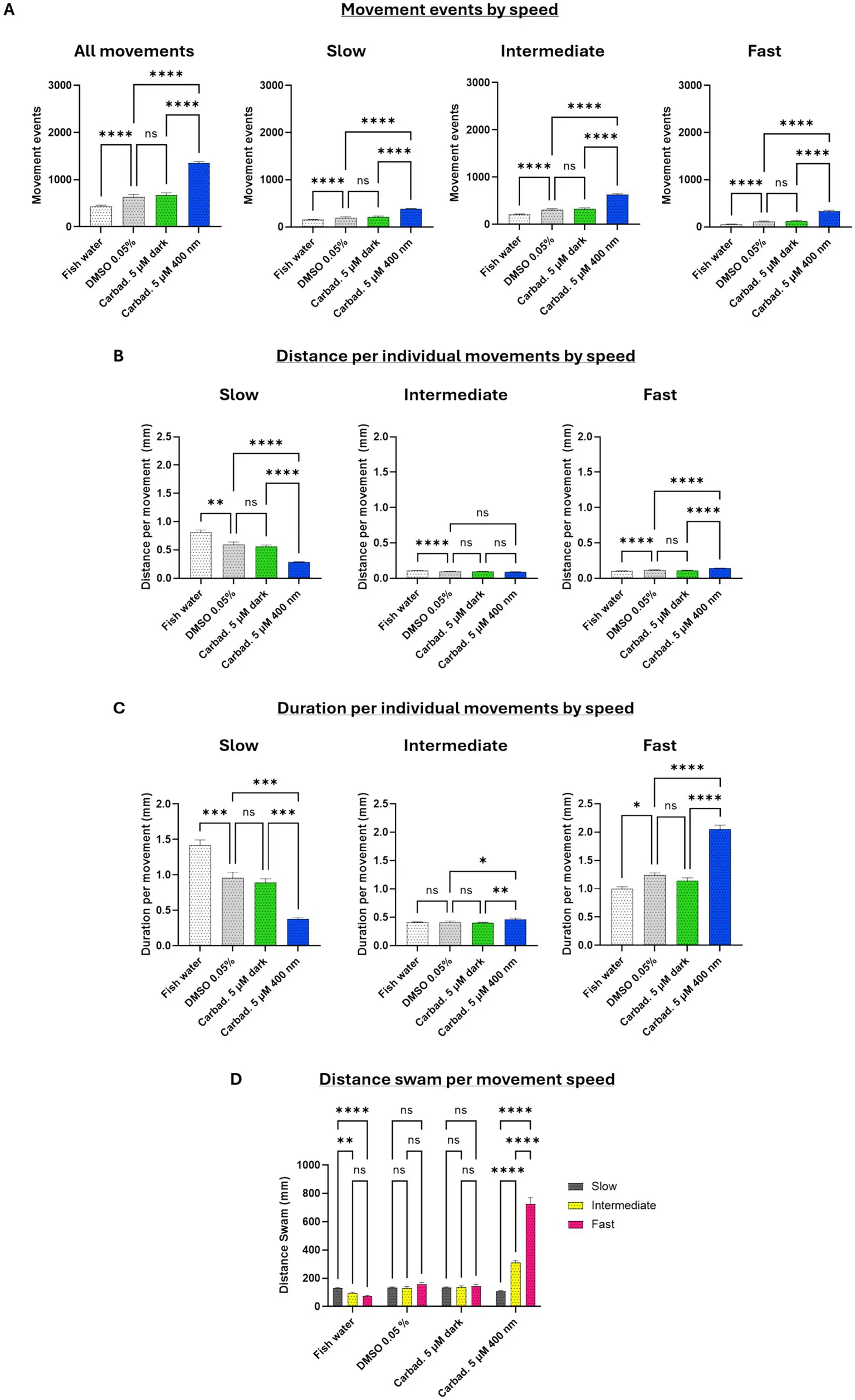
Analysis of the speed of movements during no-stimuli period of the Optomotor response assay in free swimming larvae. **(A)** Count of total movement events (i.e., every time a larva initiates a movement) and classified by speed of movement for larvae in fish water, treated with vehicle (DMSO 0.05%, *n* = 40), Carbadiazocine dark, and Carbadiazocine pre-illuminated at 400 nm (*n* = 50/group). ****p < 0.0001; ns = not significant, Mixed-effect analysis followed by Šídák’s multiple comparisons test. **(B)** Distance traveled in each slow, intermediate and fast movement for larvae in fish water, treated with vehicle (DMSO 0.05%, *n* = 40), Carbadiazocine dark, and Carbadiazocine pre-illuminated at 400 nm (*n* = 50/group). ****p < 0.0001; ***p < 0.001; **p < 0.01; *p < 0.05; ns = not significant, Mixed-effect analysis followed by Šídák’s multiple comparisons test. **(C)** Duration of each slow, intermediate and fast movement for larvae in fish water, treated with vehicle (DMSO 0.05%, *n* = 40), Carbadiazocine cis and Carbadiazocine pre-illuminated at 400 nm (*n* = 50/group). ****p < 0.0001; ***p < 0.001; **p < 0.01; *p < 0.05; ns = not significant, Mixed-effect analysis followed by Šídák’s multiple comparisons test. **(D)** Distance swam with slow, intermediate and fast movements for larvae in basal condition, treated with vehicle (DMSO 0.05%, *n* = 40), benchtop Carbadiazocine and Carbadiazocine pre-illuminated at 400 nm (*n* = 50/group). See Supplementary Figure S5 for the estimation plot. ****p < 0.0001; ns = not significant, Two-way ANOVA followed by Šídák’s multiple comparisons test.

The free-swimming OMR assay reveals that pre-illuminated Carbadiazocine profoundly alters larvae swimming patterns beyond a simple increase in locomotion. During the no-stimulus period under different treatments, we observed changes in movement duration, range, and frequency across different speeds. This hyperactivity disrupted the OMR response, causing larvae to perform irregular swims—including movements orthogonal to, and even against, the direction of the visual stripes—thereby impairing their ability to properly follow the visual cues.

Encouraged by these important differences between Carbadiazocine isomers in the free-swimming behavior, we attempted real-time photoswitching experiments (Figure 3; Supplementary Figure S6). A difficulty of these experiments is that changing light already produces behavioral effects. These ‘startle’ responses are well documented (Guggiana-Nilo and Engert, 2016; Zimmermann et al., 2018) and cannot be avoided by blinding procedures for this assay (Matera et al., 2022; Camerin et al., 2024) because OMR requires visual perception. Despite this important limitation, we observe a significant decrease in OMR correctness after irradiating the plate with 400 nm light in larvae treated with 5 μM dark-relaxed Carbadiazocine compared to untreated larvae, and these differences are reversed by 500 nm illumination (Figure 3; Supplementary Figure S6).

**Figure 3:**
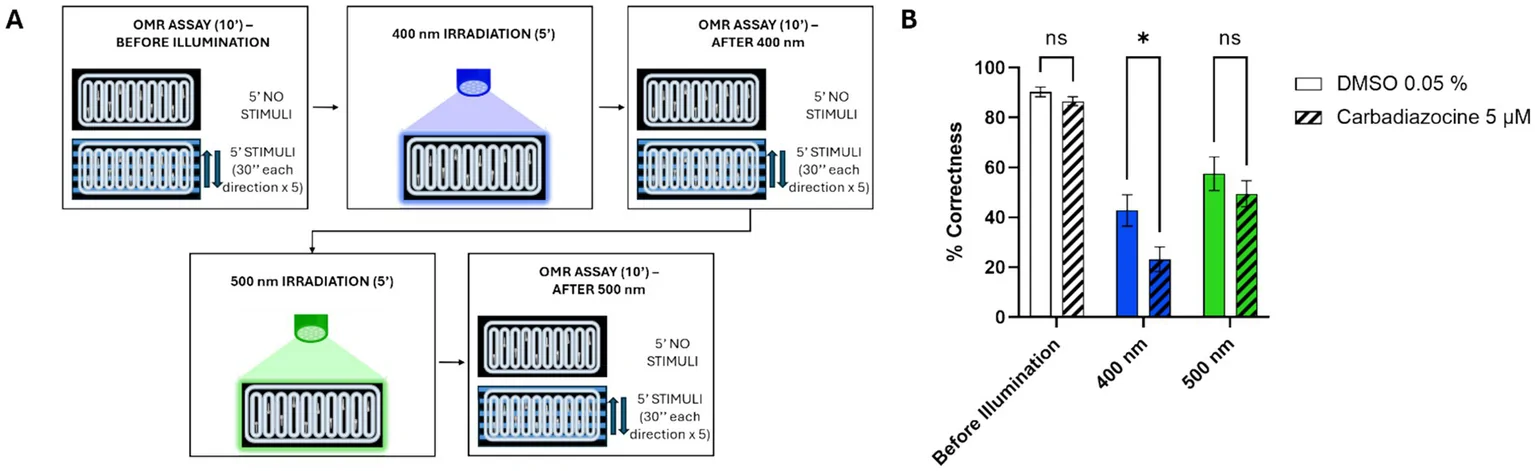
Real-time photoswitching during a free-swimming optomotor response (OMR). **(A)** Experimental design of the sequential free-swimming OMR assays performed to assess *in vivo* photoswitching of Carbadiazocine. Zebrafish larvae were treated with vehicle (DMSO 0.05%, *n* = 36) or dark-relaxed Carbadiazocine 5 μM (*n* = 38), and the OMR assay was performed sequentially at three time points: before illumination, after 5-min irradiation at 400 nm and after subsequent 5-min irradiation at 500 nm. Created in BioRender. Martínez Tambella, J. (2026) https://BioRender.com/du1x0vi. **(B)** Comparison of the OMR correctness between vehicle-treated and Carbadiazocine 5 μM treated larvae under each condition. Before illumination, there are no significant differences in correctness between groups, while after 400 nm irradiation and the resulting photoisomerization of Carbadiazocine, the correctness of the treated larvae decreases significantly compared to the vehicle control. Illumination with 500 nm light with back-isomerization to the dark-relaxed isomer of Carbadiazocine shows reversibility of the effect, recovering control-level correctness percentages. * *p* < 0.05, Two-way ANOVA with Šidák’s multiple comparisons test.

While the free-swimming OMR assay provides an integrated measurement of overall swimming behavior throughout the experimental period, it lacks the precision to analyze the larvae’s initial responses to stimulation at individual swim bouts level. To address this limitation, we employed head-fixed experiments in which the larvae’s heads are immobilized in agarose gel while the tail remains free to move, allowing for detailed analysis of individual swim bouts. Furthermore, we replaced striped patterns by an RDK as visual stimulus, which provides a more controlled and refined stimulus for probing zebrafish larval behavior (Bahl and Engert, 2020). The RDK assay was previously used by Bahl and Engert (2020) to validate experimentally the probabilistic decision-making model they developed to predict how zebrafish larvae exposed to a visual stimulus can integrate and retain sensory evidence over several seconds and provide a valuable model for investigating neural circuit and decision-making processes (Dragomir et al., 2020).

The RDK-evoked OMR assay can also benefit from the ability of turning on and off selected neurons using photopharmacology, to add granularity and facilitate the search for correlations between circuits and behavioral outcomes. Therefore, we evaluated Carbadiazocine in RDK-OMR experiments using larval zebrafish at 5–7 dpf. Larvae were embedded in transparent agarose, leaving the tail free to move in response to stimuli and leaving the mouth exposed to the medium to allow drug uptake. Excess solidified agarose was removed from the dish, as the gel can serve as a water reservoir and dilute the concentration of drug solutions. The animals were exposed to the random dot motion stimuli projected on the bottom of the arena and their behavior was tracked in real time (see SI methods) with a custom-written Python 3.7-based software package. The stimulus was designed to be perpendicular to the larvae body axis and in a closed loop with larvae movements (Figure 4). Each detected swim bout was defined as correct if the first movement of the tail followed the direction of the stimulus. Increasing percentages of dots moving in the same direction in the pattern (coherence) induce an increased accuracy in the decision-making process (correctness) and a decrease in the responding time to bout in 5–7 dpf larvae (Bahl and Engert, 2020).

**Figure 4:**
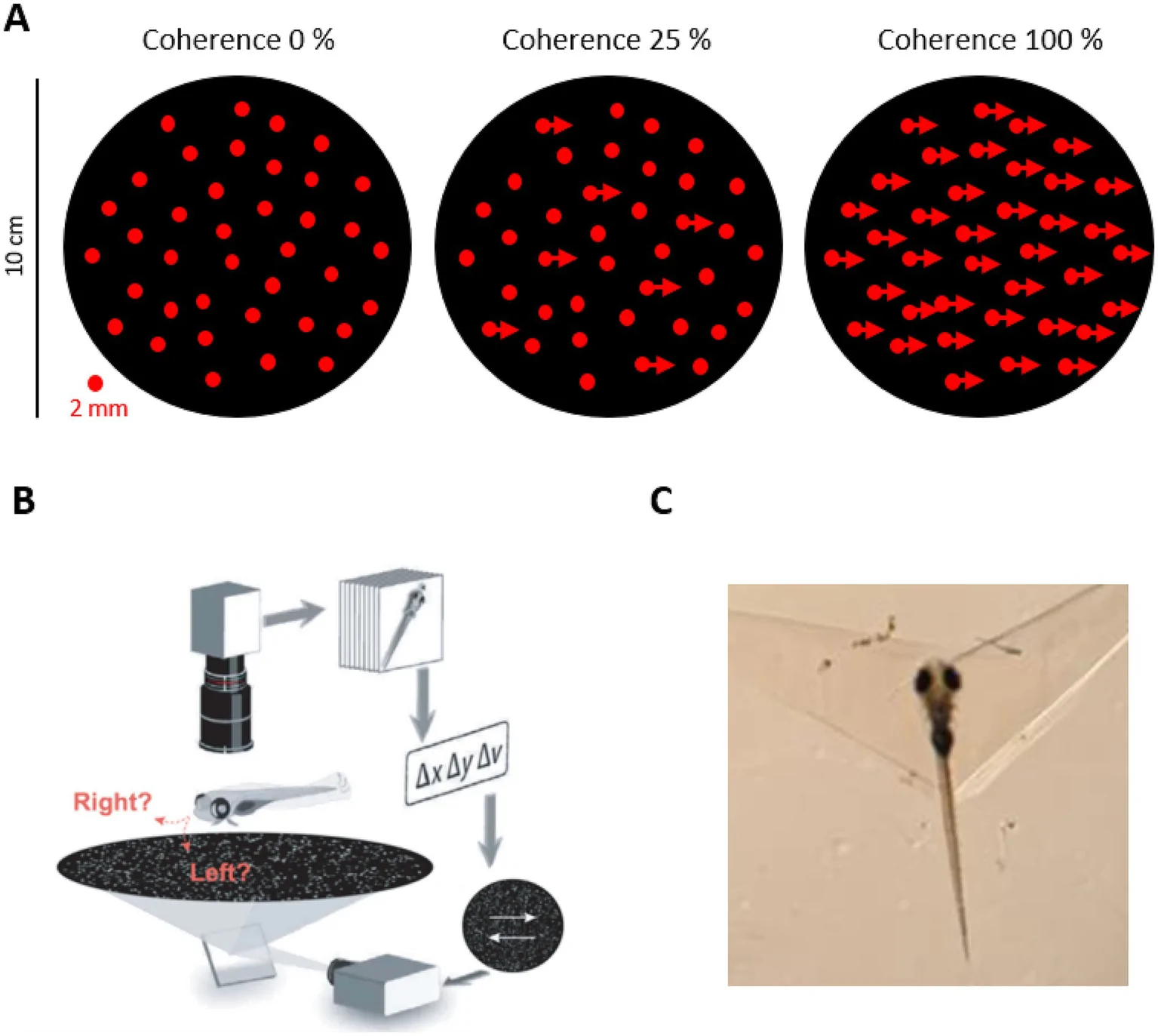
**(A)** Representation of random dot kinematogram (RDK) visual stimuli at 0, 25 and 100% coherence (e.g., rightwards) adopted from Bahl and Engert (2020). The real stimuli applied to the larvae include around 1,000 dots, each 2 mm in diameter, projected on a circular area with 10 cm diameter. The white light from the projector was filtered (filter cut-on wavelength 620 nm) to obtain red dots at wavelengths that are not interfering with the switching of Carbadiazocine. **(B)** Representation from Bahl and Engert (2020) experimental set up with the visual stimuli projected from the bottom, perpendicular to the larvae. The camera and lens from the top enables tracking tail movements with a custom-written Python 3.7-based software package. **(C)** Zebrafish larva embedded in agarose leaving free mouth and tail, respectively to allow drug uptake and tail movements in response to stimuli.

We conducted experiments to test Carbadiazocine, dividing the larvae into three groups for each experimental session: (1) fish treated with either 10 μM or 30 μM of the dark thermally relaxed isoform (100% *cis*), (2) fish treated with a solution pre-illuminated with 405 nm (50% *trans*; Camerin et al., 2024), and (3) vehicles (fish water with 0.1 and 0.3% DMSO respectively). After embedding, larvae were left to rest for at least 10 min in filtered embryo water (E3) before they were exposed to the visual stimulus for 1 h. This duration ensured sufficient trials at each coherence level, allowing to record baseline swimming activity and responsiveness to the stimuli. The fish water was then removed and changed with the prepared solutions (dark, pre-illuminated compound or vehicle respectively). After an incubation period of 20 min, the larvae underwent another hour of exposure to the random dots motion.

The swimming activity of each fish in head-fixed experiments was recorded and represented as raw traces of individual larvae tail movements (see representative examples in SI Figure S9). The traces show all tail movements, regardless of the threshold for automated bout detection and regardless of the visual stimuli. Larvae treated with either the vehicle or the dark-relaxed compound showed no change in swimming activity before and after drug administration. However, fish treated with 405 nm pre-illuminated Carbadiazocine exhibited distinct changes in behavior compared to controls: the frequency of subthreshold small swims increased, interspersed with periods of complete inactivity (Supplementary Figure S9; Supplementary Figure A), which may be due to exhaustion following hyperactivity (Camerin et al., 2024).

The small subthreshold swims induced by treatment with 405 nm pre-illuminated Carbadiazocine were below the bout detection threshold of the tracking software and were therefore not classified as swimming bouts. Lowering the bout detection threshold was not appropriate because it resulted in the detection of movements that interfered with the initiation of new visual stimulus trials (see Methods for details of the visual stimulus). Therefore, to quantify these subthreshold movements independently of bout detection, we analyzed the tail angle integral during 0% coherence trials, which showed a significant increase only upon application of pre-illuminated Carbadiazocine (Figure 5B). Moreover, the power spectrum of these subthreshold swims was analyzed, evidencing that Carbadiazocine pre-illuminated at 405 nm induced an increase in the proportion of swims with a tail movement frequency between 15 and 30 Hz (Figure 5C, analysis of normalized area under the curve AUC in Supplementary Figure S10, S11). The increased swimming frequency observed in traces and videos of head-fixed larvae (see Supplementary Figure S9; Supplementary Movies 3, 4) agrees with the formerly described increased locomotion of blinded larvae treated with 405 nm-illuminated Carbadiazocine (Camerin et al., 2024).

**Figure 5:**
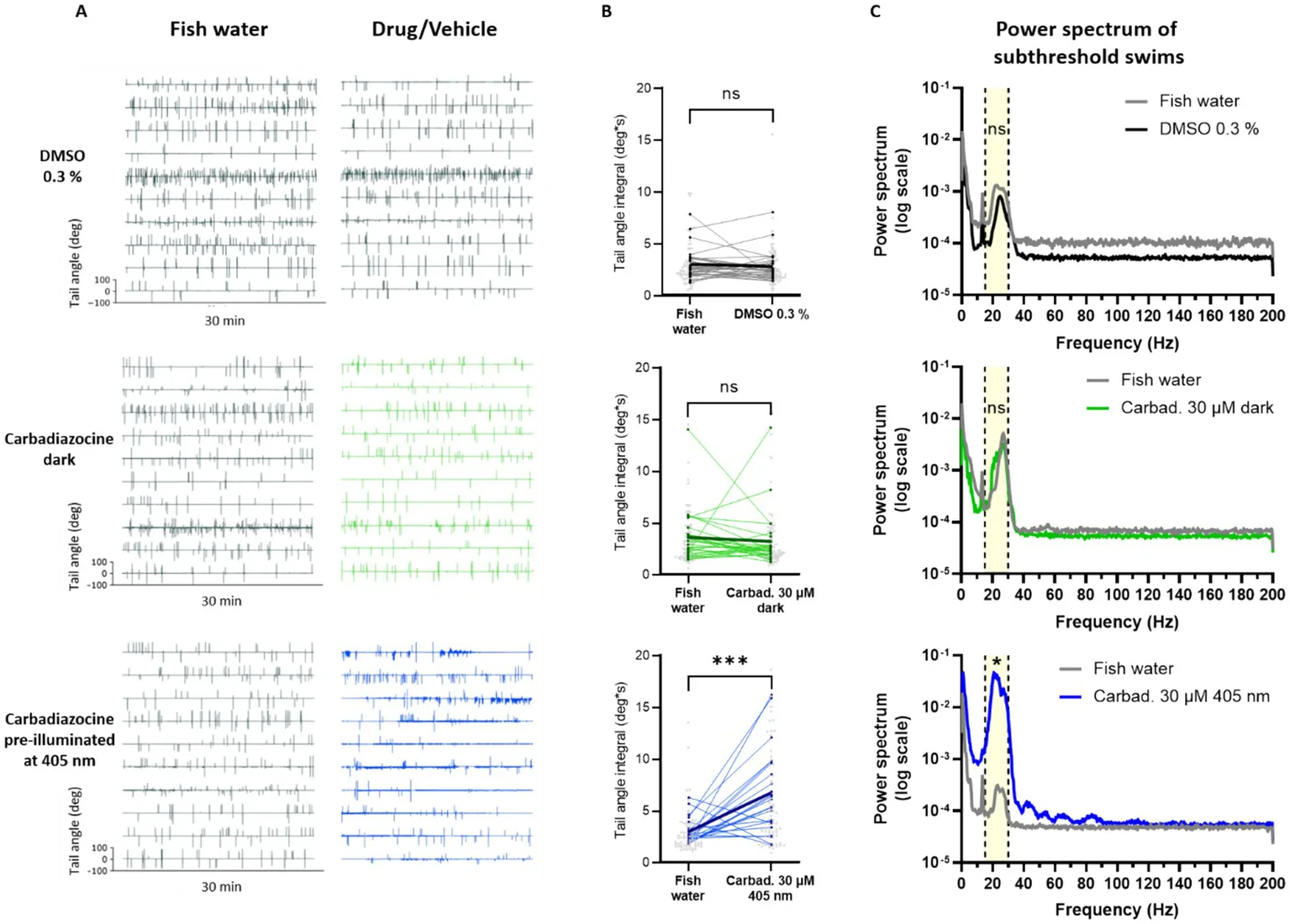
**(A)** Raw traces of tail movements from individual fish illustrating basal swimming activity in fish water and swimming activity post addition of the respective test solutions (vehicle 0.3% DMSO, Carbadiazocine dark, and Carbadiazocine pre-illuminated at 405 nm at 30 μM). **(B)** Computed tail-angle of subthreshold swims (0% coherence trials) by integrating the absolute value of the baseline-corrected traces. Wilcoxon test, *** *p* < 0.0003. Control 0.3% DMSO: *n* = 30; Carbadiazocine dark: *n* = 28, data point cut off (d.p.c.o.) = 1; Carbadiazocine pre-illuminated at 405 nm: *n* = 23, data point cut off (d.p.c.o.) = 3. **(C)** Frequency components and power spectrum of 0% coherence traces (subthreshold swims). The area under the curve of the band between 15–30 Hz was quantified and normalized to the total area under the curve (see Supplementary Figure S10, S11 for quantification). ns, Not significant; *p < 0.05 treatment vs. fish water, Wilcoxon matched pairs signed rank test.

Using this setup and analysis method, we aimed to investigate whether Carbadiazocine interfered with the ability of zebrafish larvae to integrate motion-derived sensory input and produce robust directional behavior (Bahl and Engert, 2020; Dragomir et al., 2020). We evaluated the behavior at three coherence levels (0, 25 and 100%). Experiments with 10 μM Carbadiazocine only showed significant difference for the pre-illuminated isomer at 100% coherence (see Supplementary Figure S12), possibly due to the agarose absorbing the drug and diluting it in the medium. Therefore, only the results from experiments at 30 μM were analyzed. ^[32]^We analyzed the correctness of the bouts based on the first tail deflection (see SI Methods). We observed that in larvae treated with vehicle (0.3% DMSO) or dark-relaxed Carbadiazocine 30 μM (100% *cis*), correctness increased with the coherence level, as reported for untreated larvae (Figure 6) (Bahl and Engert, 2020). In contrast, larvae treated with Carbadiazocine 30 μM pre-illuminated at 405 nm (50% *trans*) showed lower correctness in response to the visual stimuli (reduction from ~80% to ~65% for 25% coherence; from ~95% to ~80% for 100% coherence), in agreement with observations from OMR assays. Notably, the dark-relaxed isomer of Carbadiazocine did not affect performance at any coherence level (Figure 6, normalized data in Supplementary Figure S13).

**Figure 6:**
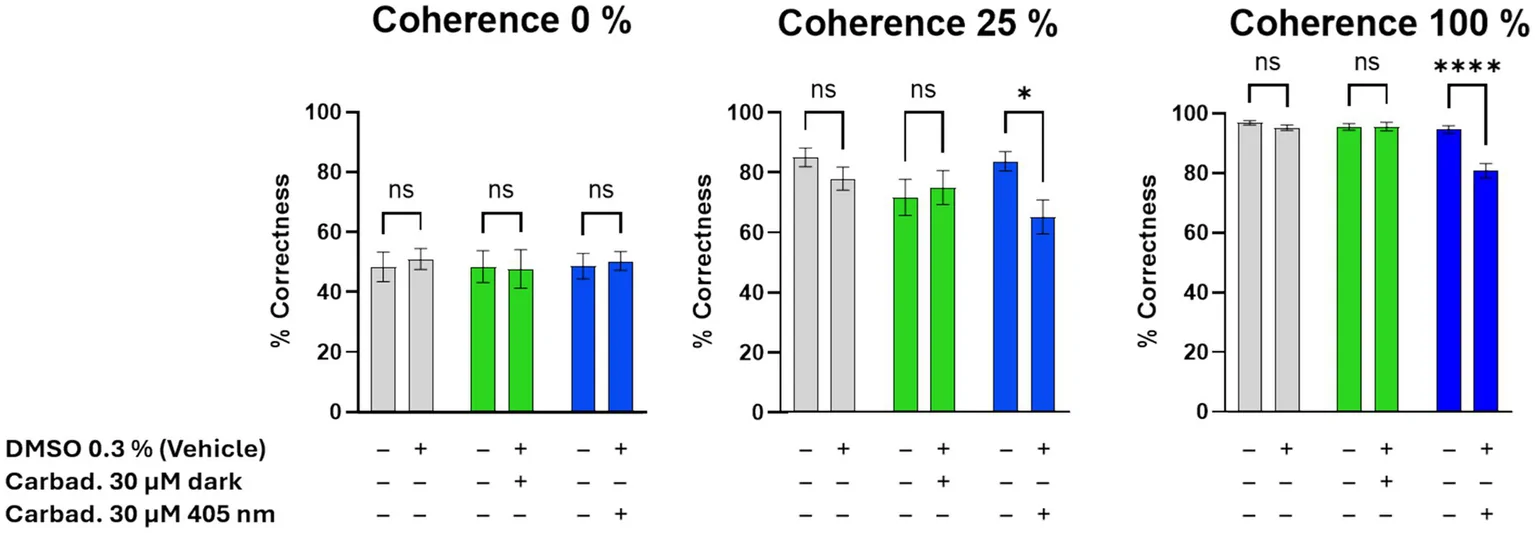
Percentage of correctness for each level of coherence (0, 25, and 100%). Correctness drops in zebrafish larvae treated with Carbadiazocine (30 μM) pre-illuminated at 405 nm in the presence of visual stimuli (25 and 100% coherence) but not with Carbadiazocine dark (30 μM). Symbols “+” and “–“indicate the presence or the absence of DMSO 0.3%, dark Carbadiazocine and Carbadiazocine pre-illuminated, respectively. **** *p* < 0.0001 Two-Way ANOVA with Šídák’s multiple comparisons test. Error bars represent S. E. M, *n* = 32/group.

Taken together, these results indicate that the dark Carbadiazocine isomer does not alter movement correctness compared to vehicle controls (0.3% DMSO) but selectively impairs performance following irradiation at 405 nm.

## Discussion

In this report we combine for the first time advanced OMR assays with photopharmacology to investigate the effects of Carbadiazocine on zebrafish sensorimotor behaviors. The two behavioral paradigms implemented in zebrafish larvae, under both head-fixed and freely swimming conditions, provide complementary approaches to characterize the photopharmacological modulation of visually guided behaviors. Compared with conventional locomotion assays, these assays offer greater behavioral resolution and enable a more detailed investigation of the sensorimotor processes. Together, the two assays provide a comprehensive assessment of visually guided behavior in zebrafish larvae, with each paradigm contributing distinct and complementary information. As reported, Carbadiazocine exerts isomer-dependent effects on locomotion that have now been profiled in detail, including the type of movements affected, their number, duration, and distance, along with the bout frequency range that is altered by the drug. Remarkably, in both assays the effects of the drug compared to the vehicle (0.05% DMSO) are only observed after illumination.

Interpreting these results requires considering the complex neural architecture of the OMR. The OMR is a multi-step visually guided behavior that engages a distributed neural circuit: visual motion is first detected by the retina and relayed via retinal arborization fields to the pretectum and optic tectum, where directional information is processed and integrated; descending motor commands are then transmitted through premotor nuclei — including the nucleus of the medial longitudinal fasciculus (nMLF) and reticulospinal neurons, with potential cerebellar modulation — to spinal cord circuits that drive tail musculature (Naumann et al., 2016). Critically, this circuit also supports the temporal accumulation of sensory evidence, a property particularly relevant when behavior is assessed over extended timescales (Bahl and Engert, 2020).

Zebrafish OMR performance is affected to a different extent in the two visually guided behavioral assays. Correctness decreases more sharply in the freely swimming paradigm (from ~65% to ~20%) than in the head-fixed paradigm (from ~95% to ~80%). This result likely arises from differences in temporal integration and movement-analysis resolution between the two approaches. While the head-fixed assay specifically evaluates whether the initial swim bout (ms scale) is aligned with the direction of dot motion, the freely swimming assay integrates locomotor responses over a 30-s stimulation period.

The relatively preserved correctness in the head-fixed condition (80% in Figure 6) suggests that the initial perception and directional decision based on sensory input remain largely intact following treatment with pre-illuminated Carbadiazocine. In contrast, when behavior is assessed over longer time scales in freely swimming larvae, the compound-induced swimming hyperactivity appears to impair sustained OMR performance — as if the animals skidded or overshot. Although many treated larvae can still initiate movement in the correct direction (see trajectories, starting and final position in Figure 1F), their subsequent trajectories often become irregular, with swimming bouts that are orthogonal to or even opposite the direction of optic flow (Figures 1C,D), suggesting a paradoxical reversal of the expected OMR response. While the mechanisms underlying this behavior remain unclear, this unxepected phenotype indicates that Carbadiazocine-induced modulation of sensorimotor circuits can disrupt the coupling between visual motion perception and sustained motor output. As a result, the accumulated ΔY displacement over the entire stimulus period no longer reflects proper tracking of the moving pattern by the larvae. Consistently, changes in the average Y coordinate upon stimulus presentation indicate that treated larvae can detect the visual stimulus but fail to follow it reliably and continuously (Figure 1C). The lower correctness observed in the freely swimming assay may therefore reflect the accumulation of these irregular non-random movements over time, amplifying subtle initial impairments into pronounced performance deficits.

In head-fixed OMR experiments, we also recorded and quantified several parameters (time to bout, number and fraction of completed trials, percentage of correctness over time, etc.; see Supplementary Figures S14–S18). In principle, these parameters offer valuable metrics to characterize the effects of photoswitchable or non-photoswitchable compounds. Although in this article only some of these parameters were significantly altered by Carbadiazocine isomers, they constitute an test suite to profile compounds with improved (photo) pharmacological properties. Combining OMR assays with a diverse library of compounds targeting different neuronal proteins may therefore provide a powerful approach to identify and characterize the neural circuits underlying complex behaviors. Previous studies have used OMR to investigate whether silencing sensory experience through chronic exposure to tricaine during early developmental stages affects the emergence of complex sensorimotor behaviors (Barabási et al., 2024). While this study focused on chronic administration—demonstrating no change in larval performance following washout of the anesthetic—we instead integrated the OMR assay with acute treatment using Carbadiazocine to induce behavioral perturbations.

Besides providing a complete characterization of the zebrafish larvae locomotor modulation by Carbadiazocine and its effects on sensorimotor behavioral assays, the results presented here show great prospects for this molecule as a tool to study neural circuits underlying different types of movement and decision-making processes. In the zebrafish larvae brain, a cluster of ~300 descending projection neurons are connected to the spinal cord (Kimmel et al., 1982). These neurons can be genetically labeled with fluorescent calcium and voltage sensors and studied with advanced imaging techniques. They exhibit distinct morphological and functional properties, enabling the coordination of various behavioral outputs. Systematically testing their activity upon Carbadiazocine administration combined with precise spatiotemporal activation using light, would provide deeper insights into the contribution of specific neuronal subpopulations within this cluster to the different movement types that are modulated by Carbadiazocine. Importantly, the reversibly photoswitchable properties of Carbadiazocine, together with the absence of behavioral effects of its dark-relaxed isomer, allow exerting precise activity patterns on neurons, making it a powerful tool for circuit analysis. Preliminary results of Figure 3 and SI Figure S6 show that these experiments are feasible despite the intrinsic behavioral effects of illumination (Camerin et al., 2024; Portugues and Engert, 2009; Zimmermann et al., 2018; Matera et al., 2022). Probably, head-fixed experiments with focalized laser scanning illumination may offer greater versatility to selectively photoswitch the circuits under study while minimizing confounding signals via the retina.

The spatial resolution of photostimulation depends on the illumination method (continuous wave or multiphoton) (Sortino et al., 2023; Izquierdo-Serra et al., 2014; Pittolo et al., 2019; Garrido-Charles et al., 2025) but it is diffraction-limited to a fraction of a micrometer for ultraviolet and visible wavelengths used in photopharmacology. It can also be limited by diffusion of the compound. In neurons, an axial resolution of ~5 μm has been achieved with a diffusible ligand (Pittolo et al., 2019) and ~1 μm on the image plane with a tethered one, allowing to control the activity of individual synapses *in vivo* (Garrido-Charles et al., 2025). Thus, combining spatiotemporal photostimulation patterns (by single- or multi-photon laser scanning or holography) with OMR-based assays will allow delineating neural circuits and understanding the corresponding mechanisms at the level of synaptic functional units. Note that, in contrast to optogenetic manipulations, which operate at the cellular scale by means of available gene promoters, photopharmacology offers to directly control endogenous proteins in their physiological context (Camerin et al., 2026) and thus leveraging their corresponding signaling pathways and downstream biological processes, including behavioral outcomes.

In summary, the combination of freely moving and head fixed OMR assays with photopharmacology and the prospects of using high resolution neuronal activity imaging and manipulation with photoswitchable drugs, together offer an opportunity to probe intact neuronal circuits underlying sensorimotor transformations in diverse animal species and wide spatiotemporal scales.

## Conclusion

In this work, we have investigated the impact of the photoswitchable compound Carbadiazocine on sensorimotor behavior and the ability to detect and follow visual stimuli of zebrafish larvae. We have analyzed the effects of the drug isomers on non-stimulated and visually guided locomotion by combining two advanced complementary assays. They allow for the classification and quantification of the movements of the larvae in response to different visual stimuli (forward-backward moving stripes and right–left moving dots at different coherence levels) and in different configurations (freely swimming and head fixed). Each assay provides extensive data on larval zebrafish locomotion, including at individual bout level with resolution of tail angles and bout frequency, and at a larger scale with distances, speeds, duration, and number of movements. Together, these assays establish a platform to evaluate the effect of photoswitchable and non-photoswitchable drugs in this animal model. The in-depth characterization of Carbadiazocine effects and its light-dependent activity posits it as a unique tool to study neural circuits underlying specific movements using patterns of photostimulation. Spatiotemporal manipulation of circuits enables to selectively perturb and probe neural computations in order to crack the intricate networks underlying behavior.

## Methods

### Zebrafish housing - head-fixed experiments

The larvae were obtained from crossing AB wild type zebrafish (*Danio Rerio*). Larvae were raised in groups of 20–30 individuals in filtered fish facility water in Petri dishes (9 cm diameter) cleaned and refilled daily. The animal development was checked every 24 h, and abnormal or unhealthy embryos/larvae were removed. The environmental conditions during the development were the following: constant temperature at 28 °C and 14 h light – 10 h dark cycles. Larvae were fed daily with paramecia from 4 dpf. Experiments were performed with larvae at age range of 5–7 dpf. Sex cannot be determined at this stage. At the end of the experiments, larvae were euthanized in tricaine methanesulfonate 0.02%. All experiments were conducted with the approval of the Harvard University Standing Committee on the Use of Animals in Research and Training.

### Zebrafish housing–free swimming experiments

Wild-type zebrafish embryos (Tupfel long-fin strain, *Danio Rerio*) were obtained from the animal facility at the Barcelona Biomedical Research Park. They were raised in darkness at 28 °C for 7 days in UV-filtered tap water, housed in Petri dishes that were cleaned and refilled daily. Embryonic development was monitored every 24 h, and any unhealthy or abnormal embryos or larvae were removed and euthanized using 0.02% tricaine methanesulfonate. All experiments and procedures complied with the European Directive 2010/63/EU.

### Visual stimulus - head-fixed experiments

Random dot motion kinematograms (RDK) were employed as visual stimuli. Although various versions of this stimulus exist (Rajananda et al., 2018), the one referenced in this work consists of a cloud of white dots (filtered, red) displayed against a black background. These stimuli was adopted from Bahl and Engert (2020) and comprised approximately 1,000 dots, each 2 mm in diameter (generated online using custom-written programs in Python 3.7 and Panda3D), projected at 60 Hz using an AAXA P300 Pico Projector onto a circular arena with a 10 cm diameter, constructed from mildly light-scattering parchment paper. The white light from the projector was filtered with Hoya R62 (cut-on WL 620 nm), 25.4 mm Diameter, 2.5 mm Thick, Colored Glass Longpass Filter to obtain red dots at wavelengths that are not interfering with the switching of Carbadiazocine. The behaviourally relevant visual field was approximately 5 cm in radius around the animal.

In the context of RDK, the term *coherence* refers to the strength of the stimulus, which can be regulated by varying the proportion of dots moving *coherently* in the same direction (Figure 4). A coherence level of 100% (the strongest stimulus) indicates that all dots move uniformly in one direction, while a coherence of 0% represents a static or flickering stimulus, where the dots flicker without directional movement. Coherence values between 0 and 100% reflect the probability that a dot follows the designated motion direction.

The random dots were projected in three different coherence levels (0, 25, 100%) each of them having two directions (−1, 1, left and right respectively). Before starting each trial, 5 s at 0% coherence were presented as a baseline stimulus (no movement, stationary flickering dots), then the stimulus was suddenly switched to one of the coherence levels, with the dots remaining stationary or translating continuously (speed: 1.8 cm s^−1^) either leftwards or rightwards, perpendicular to the animal body. Closed loop was adopted, meaning that the visual stimulus is interrupted every time a bout is detected, ending the trail. A new trial can start only if the larvae are not responding during the initial 5 s of adaptation, otherwise this baseline restarts. Each trial is lasting for a maximum of 45 s and in absence of response from the fish (timeout) a new trial was automatically initiated. Stimulus protocol: 5 s of 0% coherence, followed by 0, 25% or 100% coherence moving rightward or leftward, followed by 5 s of 0% coherence. The stimulation order was random.

Only bouts within the threshold (bout start vigor: 50, end vigor: 10, custom-made tracking software, see section SI below) are detected while smaller swims are not registered and do not interrupt the trial. Each detected swim is labeled as either correct or incorrect based on the initial tail deflection or on the integration of the full bout. If the bout turns toward the same direction as the moving dots, it is classified as correct.

Each dot, whether static or moving, had a short average lifetime of 200 ms, during which it stochastically disappeared and immediately reappeared at a random location. This design ensured that the animals could not track individual dots, avoiding potential confounds in the experiments. The dots were red (> 620 nm, filtered from white light) against a black background (average scene luminance of approximately 120 Lux, Extech Instruments Light Meter LT300).

### Visual stimulus - free swimming experiments

The stimuli consisted of 5 min in complete darkness followed by a pattern of blue and black stripes (10 in total) moving vertically at a speed of 10 revolutions per minute for 30 s in each direction (upwards and downwards) for 5 min. The stimuli was generated using OMR module in Zebrabox (Viewpoint Behavior Technology, France). To verify that the blue light projected for the visual stimuli was not photoswitching the compound, controls were run with the Carbadiazocine solutions (dark-inactive and active pre-illuminated at 400 nm), see SI Figure S7.

### Tracking and assay protocol - head-fixed experiments

Larvae were embedded in freshly made 2% agarose (UltraPure Low Melting Point Agarose, 16,520–100, Invitrogen) at approximately 35 °C, in non-treated Petri dishes (5 cm diameter). After solidifying (~ 1 min), the dish was filled with filtered facility water and the agarose around the tail and the mouth was removed leaving the minimum amount possible. The experiments were performed immediately after the embedding. The scene was illuminated from the bottom using infrared light-emitting diode (LED) panels (940 nm panel, Cop Security). Tracking was performed with a Grasshopper3-NIR camera (FLIR Systems) equipped with a Navitar Zoom 7,000 lens (18–108 mm) and an R72 long-pass filter (Hoya). Using a custom-written Python 3.7-based software package, the trail was tracked following 27 nodes along the tail. Tracking was conducted using two computers, each connected to four cameras and two projectors, enabling independent stimulation and closed-loop tracking of eight individual fish simultaneously.

Larvae were divided into three groups: (1) fish treated with 10 μM or 30 μM solution of Carbadiazocine in its dark thermally relaxed isoform (100% *cis*), (2) fish treated with Carbadiazocine pre-illuminated solution at 405 nm (50% *trans*) and (3) vehicles (fish water with 0.1 and 0.3% DMSO respectively). Experiments started at least 10 min after embedding. For all three groups of head-fixed larvae, basal swimming activity and responses to visual stimuli were recorded for 1 h in filtered facility water. This duration ensured the collection of a sufficient number of trials for each coherence level. Subsequently, the filtered facility water was removed from the Petri dish using a plastic pipette, and the respective solutions for each group were added. The larvae were incubated in the new solutions for 20 min before being exposed to the visual stimuli and tracked for one additional hour.

### Tracking and assay protocol - free swimming experiments

Free-swimming Optomotor Response (OMR) assay was performed and recorded using a Viewpoint Zebrabox videotracking instrument and Zebralab analysis software (see Figure 1; Supplementary material). Larvae were placed in 10-well plates containing 2 mL of fish water per well. The stimuli screen was below the zebrafish larvae plate inside the Zebrabox. The protocol of the assay consisted of 5 min in complete darkness followed by 5 cycles of stimuli as described above. The experiment was designed in two consecutive parts (see Figure 1A): (1) an initial phase of the recording allowed to quantify the larvae baseline swimming response to the OMR stimuli in fish water (basal). After the measurement of the basal response, larvae were left to rest for 20 min. (2) Next, 1 mL was removed from the wells and 1 mL of either vehicle (DMSO 0.1%), or Carbadiazocine 10 μM in dark conditions or pre-illuminated for 10 min with a 400 nm LED (final concentrations in well were 0.05% DMSO or 5 μM Carbadiazocine in 0.05% DMSO) was added. Immediately after the addition of the corresponding compound, the plate was placed in the Zebrabox to measure again the OMR response.

The recordings obtained from each experiment were processed through two different modules of the Zebralab software to obtain the XY coordinates of each larva and the number of movement events, swam distance and time spent swimming at different speeds, classified as slow (< 2 mm·s^−1^), intermediate (2–6 mm·s^−1^) and fast (> 6 mm·s^−1^). Data was integrated every 1 s and 30 s.

OMR assay correctness was analyzed by comparing the Delta Y coordinate for each larva during the 30 s bins of the stimuli period (10 in total, 5 with stripes moving upwards and 5 downwards). A correct response was considered when the larva Delta Y for the bin was in accordance with the direction of the stimuli (e.g., if the stripes were moving upwards and the final position of the larvae was upper than the initial) and above a Delta Y threshold established as the mean Delta Y of each larva in the absence of stimuli in 30 s bins in basal condition. Other thresholds were evaluated to assess correctness, such as an average Delta Y for each plate in basal condition in the absence of stimuli, or for control larvae, yielding the same results (see Supplementary Figure S8).

In Real-Time photoswitching experiments, the protocol consisted of 5 min in complete darkness followed by 5 min of stimuli as described above. Immediately after, 400 nm light was irradiated from above the plate for 5 min, followed by 5 min of darkness and 5 min of visual stimuli (moving stripes). Finally, 500 nm light was irradiated from above the plate for 5 min again, followed once more by 5 min of darkness and 5 min of moving stripes. Correctness was evaluated for the three stimuli periods: before illumination, after 400 nm and after 500 nm.

### Statistical analysis

All statistical tests and graphical representations were performed using Graphpad Prism 9.4.1 for Windows.^1^ Estimation plots were created using DABEST Python library. To evaluate the effects of the different treatments in free-swimming and head-fixed assays, mixed-effects analysis, repeated measures one-way ANOVA or two-way ANOVA was used depending on the experimental design. Šídák’s multiple comparisons test was employed for pre-selected pairwise comparisons, whereas Tukey’s multiple comparisons test was chosen when all available pairwise comparisons were studied. For two group comparisons, Wilcoxon matched-pairs signed rank test was utilized.

## Data Availability

The datasets presented in this study will be made available by the authors upon reasonable request.

## Footnotes

1 GraphPad Software, Boston, Massachusetts USA, www.graphpad.com
