# Supplementary material for: Remote control of wildtype zebrafish optomotor response with a photoswitchable drug
Source: Front Behav Neurosci. 2026 Oct 1;20:1916443. doi: 10.3389/fnbeh.2026.1916443 (PMC13668874; doi:10.3389/fnbeh.2026.1916443)
Supplement: Supplementary file 1 [file Data_Sheet_1.DOCX]

SUPPLEMENTARY INFORMATION (SI)

**Remote Control of Wildtype Zebrafish Optomotor Response with a Photoswitchable Drug**

Luisa Camerin ^1, 2, 3, #^, Joaquín Martínez-Tambella ^1, 2, 3, #^, Gregor Schuhknecht ^4, %^, Vickie M. Wang ^4,5^, Kumaresh Krishnan ^4, ¥^, Paula Pflitsch ^4, &^, Florian Engert ^4, *^, Pau Gorostiza ^1, 2, 6, *^

^1^ Institute for Bioengineering of Catalonia, Nanoprobes & Nanoswitches, Barcelona, Spain

^2^ Network Biomedical Research Institute in Bioengineering, Biomaterials, and Nanomedicine (CIBER-bbn), Madrid, Spain

^3^ University of Barcelona, Barcelona, Spain

^4^ Faculty of Arts and Sciences, Harvard University, Department of Molecular and Cellular Biology, Cambridge, Massachusetts, United States

^5^ Department of Neurobiology, Harvard Medical School, Boston, Massachusetts, United States

^6^ Catalan Institution of Research and Advanced Studies (ICREA), Barcelona, Spain

\# Equivalent contribution

% Present address: Max Planck Institute for Brain Research, Frankfurt am Main, Germany

¥ Present address: Howard Hughes Medical Institute (HHMI), Cambridge, Massachusetts, United States

& Present address: Leibniz Institute for Neurobiology, Magdeburg, Germany

* Correspondence: florian@mcb.harvard.edu (F. E.), pau@icrea.cat (P. G.)

Keywords: Optomotor response (OMR), Circuit perturbation, Sensorimotor behavior, Photopharmacology, Photostimulation, Neuromodulation, Optogenetics, Na_V_ voltage-gated sodium channels.

**TABLE OF CONTENTS**

[1	Additional analysis for free-swimming larval zebrafish	3](#_Toc236221172)

[2	Additional analysis for head-fixed larval zebrafish	10](#_Toc236221173)

# 1 Additional analysis for free-swimming larval zebrafish

**Figure S1:** Optomotor response assay Y coordinate through time for larvae in basal condition, treated with vehicle (DMSO 0.05 %), Carbadiazocine dark 5 µM and 400 nm pre-illuminated Carbadiazocine 5 µM, here represented as stacked lines. Horizontal dotted lines represent the central position of the well, whereas vertical dotted lines represent the timepoint where the stimuli direction changes, indicated by the arrows. Treatment with 400 nm pre-illuminated Carbadiazocine affects the capability of following the stripes direction correctly.

**Figure S2:** Estimation statistics plot for the correctness percentage in the free-swimming optomotor response assay for larvae treated with vehicle (DMSO 0.05 %), Carbadiazocine dark and Carbadiazocine pre-illuminated at 400 nm, represented as bar plots in Figure 1.E.

**Figure S3:** Count of total movement events (i.e. every time a larva initiates a movement) and classified by speed of movement for larvae in basal condition, treated with vehicle (DMSO 0.05 %), Carbadiazocine dark and Carbadiazocine pre-illuminated at 400 nm. Smooth bars represent the count for the complete experiment and striped bars during the stimuli period. Data represented as mean ± S.E.M. ****p<0.0001; ***p<0.001; **p<0.01; *p<0.05; ns = not significant, Mixed-effect analysis followed by Šidák’s multiple comparisons test.

**Figure S4:** Total swam distances and classified by speed of movement for larvae in basal condition, treated with vehicle (DMSO 0.05 %), Carbadiazocine dark and Carbadiazocine pre-illuminated at 400 nm. Smooth bars represent the swam distances for the complete experiment, dotted bars during the no-stimuli period and striped bars during the stimuli period. Data represented as mean ± S.E.M. ****p<0.0001; **p<0.01; *p<0.05; ns = not significant, Mixed-effect analysis followed by Šidák’s multiple comparisons test.

**Figure S5:** Estimation statistics plot for the distance swam at slow, intermediate or fast speed by larvae treated with with vehicle (DMSO 0.05 %), Carbadiazocine dark and Carbadiazocine pre-illuminated at 400 nm, represented as bar plots in Figure 2.D.

**Figure S6:** Real-time photoswitching during a free-swimming optomotor response (OMR) assay. Correctness was assessed before illumination, after 5-minute irradiation at 400 nm and following 5-minute irradiation at 500 nm sequentially (see Methods for details). **A)** Correctness of the OMR for vehicle-treated larvae before and after irradiation with 400 nm and 500 nm light. Light itself exerts effects on larvae behavior that affect their subsequent performance in the assay, with a more significant effect from 400 nm light. N = 36, **** p < 0.0001; * p < 0.05 Repeated measures One-way ANOVA with Šidák’s multiple comparisons test. **B)** Correctness of the OMR for larvae treated with Carbadiazocine dark 5 µM before and after irradiation with 400 nm and 500 nm light. As observed for vehicle-treated larvae, the correctness is affected by the light. N = 38, **** p < 0.0001; *** p < 0.001 Repeated measures One-way ANOVA with Šidák’s multiple comparisons test.

**Figure S7:** HPLC photostationary quantification. **A)** Dark Carbadiazocine at 5 µM remains 100% cis upon exposure to blue stripes visual stimuli. **B)** Carbadiazocine 5 µM pre-illuminated at 400 nm does not significantly reduce its *trans* % isomer upon exposure to blue stripes visual stimuli (41% vs 38%).

**Figure S8:** Correctness in the Optomotor response assay in free-swimming larvae setting different thresholds for correctness. The left panel shows the percentages of correctness setting as threshold for a positive response the average vertical distance travelled by the whole plate (ten fishes) during the no stimuli period 30 seconds bins in basal condition, while for the right panel the threshold was set as the average vertical distance travelled by each larva as its own control during the no stimuli period 30 seconds bins in basal condition. Data represented as mean ± S.E.M. ****p<0.0001; ***p<0.001; **p<0.01; *p<0.05; ns = not significant, Mixed-effect analysis followed by Šidák’s multiple comparisons test.

# 2 Additional analysis for head-fixed larval zebrafish

**Figure S9:** Raw traces recording the tracked swimming activity for each head-fixed larva (n=32). During the recordings the larvae were exposed to the visual stimuli, although it is not integrated into the row traces graphs. “Basal” graphs correspond to the performance in filtered fish water, “post” graphs correspond to the performance after the addition of the treatment solutions. Upper lines indicate “right” tail deflection, lower lines indicate “left” tail deflection **A)** Basal and post traces for vehicle (0.3% DMSO). **B)** Basal and post traces for the solution of dark-adapted Carbadiazocine isoform at 30 µM. **C)** Basal and post traces for the solution of Carbadiazocine 30 µM pre-illuminated at 405 nm.

**Figure S10:** Normalized Area under the curve (AUC) of the power spectrum of subthreshold swims shown in Figure 5.C for head-fixed larvae treated with vehicle 0.3% DMSO (dark grey), Carbadiazocine dark at 30 µM (green) and Carbadiazocine 30 µM pre-illuminated at 405 nm (blue). AUC for the 15-30 Hz frequency band was normalized by the total AUC of the power spectrum. The contribution of this frequency movements is significantly increased upon treatment with Carbadiazocine pre-illuminated at 405 nm. Data is represented as mean ± standard deviation. *p<0.05; ns = not significant, Wilcoxon matched pairs signed rank test.

**Figure S11:** Normalized Area under the curve (AUC) of the power spectrum of subthreshold swims shown in Figure 5.C for head-fixed larvae before each treatment. AUC for the 15-30 Hz frequency band was normalized by the total AUC of the power spectrum, and the values in Fish water before the addition of each treatment (vehicle – DMSO 0.05 %, Carbadiazocine 30 µM dark or Carbadiazocine 30 µM pre-illuminated at 405 nm) show no significant differences among groups (One-way ANOVA with Tukey’s multiple comparisons test).

The experiment with Carbadiazocine at 10 μM only showed significance for the pre-illuminated isomer at 100% coherence, most likely due to the dilution of the solution below the effective concentration caused by the presence of agarose.

**Figure S12:** Results for correctness for zebrafish larvae treated with Carbadiazocine at 10 μM. **A)** Percentage of correctness for each level of coherence (0%, 25% and 100%). Performance was first assessed in fish water, and secondly after treatment (vehicle 0.1% DMSO, Carbadiazocine dark 10 μM and Carbadiazocine 10 μM pre-illuminated at 405 nm, n = 32/group). Significant drop in correctness was only observed for 100% coherence with larvae treated with a solution of Carbadiazocine pre-illuminated at 405 nm. Symbols “+” and “–“ indicate the presence or the absence of DMSO 0.1%, dark Carbadiazocine and Carbadiazocine pre-illuminated respectively. * p<0.05 Two-Way ANOVA with Šídák’s multiple comparisons test. Error bars represent S.E.M. **B)** Normalized percentage of correctness. For each level of coherence (0%, 25% and 100%) and for each respective treatment (vehicle DMSO 0.1%, Carbadiazocine dark 10 μM and Carbadiazocine 10 μM pre-illuminated at 405 nm) the data were normalized to the correctness at the basal performance in fish facility water. One-Way ANOVA with Tukey’s multiple comparison test, ns = not significant. Error bars represent S.E.M, n=32/group.

Data from the correctness in head-fixed experiments were normalized to their respective basal performance for each coherence level (0%, 25% and 100%) and treatment (vehicle 0.3% DMSO, dark Carbadiazocine 30 μM and 405 nm pre-illuminated Carbadiazocine 30 μM). A significant drop in the percentage of correctness was observed for coherence 100% for pre-illuminated Carbadiazocine compared to vehicle and dark Carbadiazocine.

**Figure S13:** Normalized percentage of correctness. For each level of coherence (0%, 25% and 100%) and for each respective treatment (vehicle DMSO 0.3%, Carbadiazocine dark 30 μM and Carbadiazocine 30 μM pre-illuminated at 405 nm) the data were normalized to the correctness at the basal performance in fish facility water. **** p<0.0001 and ** p=0.0011 One-Way ANOVA with Tukey’s multiple comparison test. Error bars represent S.E.M, n=32/group.

The following parameters were analysed and represented in the graphs below: time to bout, number and fraction of completed trials, percentage correct over time. We considered these results less representative compared to the correctness.

The interbout-intervals (time to bout) were shortened with 405 nm pre-illuminated Carbadiazocine compared to vehicle and dark compound. This behaviour can be observed from the recorded traces or the recorded video from the camera, but it is not fairly represented in “time to bout”. In fact, quantifying this parameter cannot be done with accuracy considering that the tracking software only detected as bouts the tail movements above the manually pre-set threshold (see Methods). Larvae exposed to 405 nm pre-illuminated Carbadiazocine exhibited continuous small swims rather than distinct bouts. Given the design of the visual stimuli, counting these small swims as bouts would prevent new trials from starting, meaning the animals would only experience the initial 5 seconds of the 0 % coherence baseline. As a result, in the time-to-bout quantification, all data related to small swims are lost, and only detected bouts that surpass the threshold are recorded. For this reason, we do not consider the analysis of this parameter to be fully representative.

**Figure S14:** Percentage of correctness for the three levels of coherence (0-25-100%) and for treatments (vehicle 0.3% DMSO, dark-adapted Carbadiazocine dark 30 µM and Carbadiazocine 30 µM pre-illuminated at 405 nm). “Basal” graphs correspond to the performance in filtered fish water, “post” graphs correspond to the performance after the addition of the respective treatment solutions. Blue dots represent the average % of correctness (in successful trials) for each larva. Colored lines are connecting the average performance of each larva among the different levels of coherence. Black dots show the average % of correctness among all the larvae, for each level of coherence.

**Figure S15:** Time to first bout (s) for the three level of coherence (0-25-100%) and for the treatments (vehicle 0.3% DMSO, dark-adapted Carbadiazocine dark 30 µM and Carbadiazocine 30 µM pre-illuminated at 405 nm). “Basal” graphs correspond to the performance in filtered fish water, “post” graphs correspond to the performance after the addition of the treatment solutions. Blue dots represent the average time to bout (in successful trials) for each larva. Grey dots represent each data point. Coloured lines are connecting the average performance of each larva among the different levels of coherence. Black dots show the average time to bout among all the larvae, for each level of coherence.

**Figure S16:** Number of completed trials for the three level of coherence (0-25-100%) and for the three treatments used (vehicle 0.3% DMSO, dark-adapted Carbadiazocine dark 30 µM and Carbadiazocine30 µM pre-illuminated at 405 nm). “Basal” graphs correspond to the performance in filtered fish water, “post” graphs correspond to the performance after the addition of the treatment solutions. Blue dots represent the average number of completed trials for each larva. Colored lines are connecting the average performance of each larva among the different levels of coherence. Black dots show the average number of completed trials among all the larvae, for each level of coherence.

**Figure S17:** Fraction of completed trials (% completed trials/total trials) for the three level of coherence (0-25-100%) and for the three treatments used (vehicle 0.3% DMSO, Carbadiazocine dark 30 µM and Carbadiazocine 30 µM pre-illuminated at 405 nm). “Basal” graphs correspond to the performance in filtered fish water, “post” graphs correspond to the performance after the addition of the treatment solutions. Blue dots represent the average fraction of completed trials for each larva. Colored lines are connecting the average performance of each larva among the different levels of coherence. Black dots show the average fraction of completed trials among all the larvae, for each level of coherence.

**Figure S18**: Percentage correct over time (%) for the three level of coherence (0-25-100%) and for the treatments used (vehicle 0.3% DMSO, Carbadiazocine dark 30 µM and Carbadiazocine 30 µM pre-illuminated at 405 nm). “Basal” graphs correspond to the performance in filtered fish water, “post” graphs correspond to the performance after the addition of the treatment solutions. Light red line: 100% coherence; Dark red line: 25% coherence; Black line: 0% coherence. The length of the stimuli has been divided into bin times (0-4 sec, 4-12 sec, 12-20 sec, 20-28 sec and 28-36 sec). For each bin time it is shown the average % of correctness (dots) for the larvae responding to the stimuli during each bin time frame, for each level of coherence.
